# Perivascular Inflammation in Pulmonary Arterial Hypertension

**DOI:** 10.3390/cells9112338

**Published:** 2020-10-22

**Authors:** Yijie Hu, Leon Chi, Wolfgang M. Kuebler, Neil M. Goldenberg

**Affiliations:** 1 Keenan Research Centre for Biomedical Science, St. Michael’s Hospital, Toronto, ON M5B1W8, Canada; yijie.hu@yahoo.com; 2 Department of Cardiovascular Surgery, Research Institute of Surgery, Daping Hospital, Army Medical University, Chongqing 400042, China; 3 Department of Physiology, University of Toronto, Toronto, ON M5B1W8, Canada; leon.chi@mail.utoronto.ca; 4 Departments of Physiology and Surgery, University of Toronto, Toronto, ON M5B1W8, Canada; 5 Institute of Physiology, Charité Universitäts Medizin Berlin, 10117 Berlin, Germany; 6 Departments of Physiology and Anesthesiology and Pain Medicine, University of Toronto, Toronto, ON M5B1W8, Canada; neil.goldenberg@sickkids.ca; 7 Department of Anesthesia and Pain Medicine, Program in Cell Biology, The Hospital for Sick Children, Toronto, ON M5B1W8, Canada

**Keywords:** pulmonary hypertension, inflammation, vascular remodeling

## Abstract

Perivascular inflammation is a prominent pathologic feature in most animal models of pulmonary hypertension (PH) as well as in pulmonary arterial hypertension (PAH) patients. Accumulating evidence suggests a functional role of perivascular inflammation in the initiation and/or progression of PAH and pulmonary vascular remodeling. High levels of cytokines, chemokines, and inflammatory mediators can be detected in PAH patients and correlate with clinical outcome. Similarly, multiple immune cells, including neutrophils, macrophages, dendritic cells, mast cells, T lymphocytes, and B lymphocytes characteristically accumulate around pulmonary vessels in PAH. Concomitantly, vascular and parenchymal cells including endothelial cells, smooth muscle cells, and fibroblasts change their phenotype, resulting in altered sensitivity to inflammatory triggers and their enhanced capacity to stage inflammatory responses themselves, as well as the active secretion of cytokines and chemokines. The growing recognition of the interaction between inflammatory cells, vascular cells, and inflammatory mediators may provide important clues for the development of novel, safe, and effective immunotargeted therapies in PAH.

## 1. Introduction

**Figure 1:**
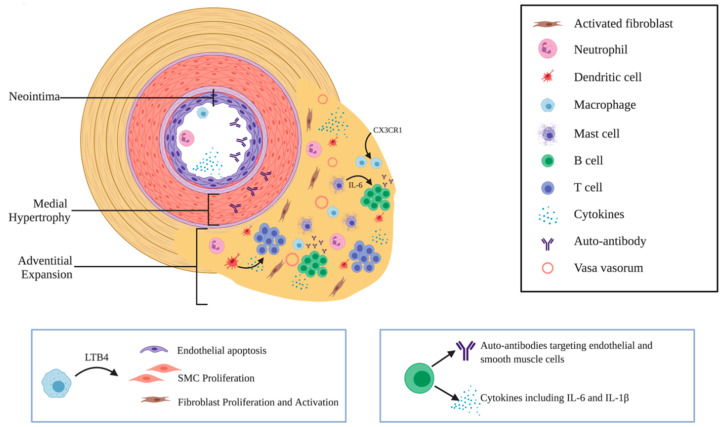
Vascular Remodeling in PAH is driven by inflammation. Pulmonary vascular lesions in PAH patients exist in an extremely inflamed micro-environment. See text for details. Note the changes to each layer of the vessel wall, including neointimal formation, medial hypertrophy, and adventitial expansion. Adventitial fibroblasts become activated and secrete cytokines and chemokines to recruit immune cells to the vessel wall via an expanded vasa vasorum. The activation of these recruited immune cells results in the further recruitment of cellular mediators as well as the secretion of cytokines, autoantibodies, and other soluble molecules. Adapted from Rabinovitch et al. *Circulation Research* 115: 165–175 (2014). Boxes highlight the actions of macrophage LTB4 (Section 2.2.1) and B cells (Section 2.3.5)**.**

Pulmonary hypertension (PH) is a devastating vascular disease characterized by remodeling of the small pulmonary arteries, elevated pulmonary artery pressure, and subsequent development of right heart failure. Pulmonary arterial hypertension (PAH; World Health Organization Group 1) represents a specific subset of this disease that is focused on the lung vasculature, and it is the subject of this review. While debate exists regarding the specific correlations to human disease in rodent models, for simplicity, data derived from animal models in this review will still be referred to as having “PAH”. Recently accumulating evidence from preclinical and clinical PAH studies have highlighted the role of inflammation in the development of the disease. At first, it was noticed that some inflammatory conditions such as connective tissue diseases are associated with an increased incidence of PAH. Next, in lung biopsies from PAH patients, virtually all lineages of inflammatory cells were detected in proximity to the remodeled pulmonary vasculature, mainly consisting of macrophages, mast cells, T lymphocytes, B lymphocytes, dendritic cells, and neutrophils (Figure 1) [1,2]. High levels of various cytokines and chemokines were confirmed in PAH animal models and PAH patients, as were the presence of autoantibodies. Importantly, the extent of this perivascular inflammation in PAH correlates with pulmonary hemodynamics, vascular remodeling, and clinical outcome [1]. Consequentially, treatments targeting inflammation and auto-immunity are currently the focus of several clinical trials in PAH patients [3]. However, the exact role of inflammation and immunity in PAH—and specifically, whether or not it is a cause, a promoter, or a downstream bystander of the disease—is poorly understood and remains a topic of considerable controversy.

Indeed, it was long thought that inflammation occurred as a secondary event during PAH pathogenesis, given that proliferating pulmonary vessel cells could secret inflammatory mediators. Yet, emerging evidence suggests that inflammation may in fact play a causal role in the development of PAH. However, many fundamental questions still remain unanswered: Is the inflammatory process nonspecific or rather directed against specific antigens? Where does this response begin—“inside–out” from endothelial cells (ECs) to the media and adventitia, or “outside–in” from the adventitia to the EC [4]?

In this review, we will address these key issues from three angles: We will discuss (A) inflammatory mediators and their effects on pulmonary vascular remodeling; (B) inflammatory/immune cells and their products in PAH; and (C) phenotypic changes in vascular cells and their feedback into the inflammatory and immune responses. Understanding the role of inflammation and immunity in PAH is not only of academic but more importantly of direct clinical interest, as a greater understanding of this interaction is expected to facilitate the evolution of new targeted therapies for this devastating disease.

## 2. Inflammatory Mediators and their Effects on Vascular Remodeling

### 2.1. Cytokines

#### 2.1.1. IL-1β

Interleukin-1β (IL-1β) is a key cytokine released in response to inflammasome activation and is an important mediator of the inflammatory response. Elevated serum levels of IL-1β have been detected in PAH patients and correlate with worse outcome [5,6]. IL-1β may in part be released from infiltrating neutrophils and T cells in diseased pulmonary vessels, as evidenced by positive staining for key components of the inflammasome system, namely Nod-like receptor family pyrin domain containing 3 (NLRP3) and apoptosis-associated speck-like protein containing a caspase-recruitment domain (ASC) within these cells in chronic hypoxia-induced PAH mice [7]. Mice deficient in ASC did not increase IL-1β when exposed to hypoxia, and they also had significantly lower right ventricular systolic pressure (RVSP) as compared to wild type [7]. See Table 1 for a brief overview of the rodent models discussed in this review.

Experimental research has shown that inhibiting IL-1β and inflammasome signaling can be an effective therapeutic avenue for PAH. Treatment with Anakinra, an IL-1β receptor (IL-1βR) antagonist, attenuated the development of PAH in monocrotaline (MCT)-treated rats [8]. Similarly, knockout of IL-1βR or the molecular adaptor myeloid differentiation primary response protein 88 (MyD88) in mice prevented against hypoxia-induced PAH [9]. Thus, in the context of PAH, neutralizing IL-1β, inhibiting IL-1β signaling, or inhibiting the upstream pathways that govern IL-1β release may be effective for mitigating disease progression.

As a potential mechanism of action, IL-1β may directly regulate the vasoconstriction and remodeling of pulmonary arteries. In pulmonary artery smooth muscle cells (PASMC), prostacyclin regulates vasodilation and has an anti-proliferative effect. This vasodilatory effect is mediated via the second messenger cyclic adenosine monophosphate (cAMP). IL-1β attenuates the conversion of ATP to cAMP in PASMC via downregulating adenylyl cyclase [10]. In addition, IL-1β could regulate PASMC growth via the IL-1R1/MyD88 pathway [11]. In line with this view, marked IL-1R1 and MyD88 expression with predominant smooth muscle cell (SMC)s immunostaining was found in lungs from patients with idiopathic PAH and mice with hypoxia-induced PAH [9].

A pilot study evaluating the safety and feasibility of anakinra for treatment of PAH was recently completed [12]. Six patients completed the study without any serious adverse events, and there was some improvement in the biomarkers and symptoms of heart failure [12]. These encouraging data will form the basis of a larger trial. Of note, this pilot study excluded patients with connective tissue disease or autoimmune disease, and it is possible that such patients would derive even greater benefit from such a treatment. Indeed, patients with connective tissue disease-associated PAH (CTD-PAH) have been shown to benefit substantially from anti-inflammatory treatments, even in trials where other groups have not. Perhaps owing to the unique pathophysiology of CTD-PAH, which clearly has a basis in dysregulated immunity, both the overall outlook and response to therapy in these patients differ from those with idiopathic disease (reviewed in [13]). Specifically in patients with systemic lupus erythematosus (SLE), anti-inflammatory treatment has yielded impressive results [14]. As such, this population merits intensive study in future trials.

**Table 1:** Overview of rodent models of pulmonary hypertension. Four of the most commonly employed rodent models are listed along with the general extent of pulmonary inflammation observed. Of note, mouse models in general exhibit less severe disease than rat models, and hypoxic pulmonary hypertension (PH) in mice is entirely reversible on return to normoxia. For a detailed examination of animal models of PH beyond the scope of this review, please see [15,16,17].

Model	Severity	Inflammation	Notes	Refs
Chronic hypoxic mouse	Mild	-Early macrophage infiltration -Requires eicosanoids -Aggravated by IL-6	-Reversible	[18]
Sugen-hypoxia mouse	Mild-moderate	-No significant pulmonary infiltration seen	-Slower to reverse than hypoxia alone	[19]
Monocrotaline rat	Severe	-Severe inflammation of lungs	-Also significant extrapulmonary inflammation	[20]
Sugen-hypoxia rat	Severe	-Closest approximation of human disease in rodents -Most immune lineages seen in lung vascular lesions	-Irreversible, plexiform angiopathy	[21]

#### 2.1.2. IL-6

Il-6 is a pleiotropic cytokine that is known to play a critical role in the progression of PAH. Plasma IL-6 levels are elevated in both patients and animal models of PAH [5,6,22,23]. Circulating IL-6 levels among PAH patients can be a useful prognostic marker, as several studies have shown a strong inverse correlation between serum IL-6 levels and long-term survival outcomes [6,24]. Although serum IL-6 levels may be poor predictors of hemodynamics in patients with PAH, strong correlations between IL-6 levels and RV dysfunction have been shown [23].

Experimental research over the past two decades has provided critical insights regarding the importance of IL-6 in the development of PAH. Fundamentally, the transgenic overexpression of IL-6 in the lungs of mice was sufficient to drive the development of mild PAH [25]. IL-6 overexpression in combination with hypoxia treatment in these mice resulted in a severe increase in RVSP and distal vascular remodeling similar to that seen in patients with severe PAH [25]. Likewise, the administration of recombinant human IL-6 produces a similar effect in mice, whereas IL-6 knockout protects against the development of hypoxia-induced PAH [26,27]. In addition, a more recent study showed that PASMC derived from patients with idiopathic PAH have upregulated membrane-bound IL-6 receptors (IL-6R) [28]. The overexpression of IL-6R promoted an anti-apoptotic phenotype in PASMCs of patients with idiopathic PAH (iPAH), but not in controls. Transgenic mice deficient in IL-6R in vascular smooth muscle are protected against the development of PAH, whereas the administration of an IL-6R specific antagonist reversed experimental PAH in two rat models [28]. At present, an open-label study of the IL-6R antagonist tolicizumab for the treatment of pulmonary arterial hypertension (TRANSFORM-UK) is running, and results are expected soon [29].

There are likely multiple cellular sources of IL-6 release in PAH. Recent studies have suggested that IL-6 can be produced by the pulmonary vasculature in PAH [28,30,31]. In particular, PASMC may be a source of IL-6, with IL-6 concentrations in conditioned media, as well as IL-6 gene expression, being significantly higher in PASMC than pulmonary artery endothelial cells (PAEC) in PAH cell lines [31]. Moreover, it has been demonstrated that pulmonary mast cells are a critical source of IL-6 production in two rat models or PAH and that mast cell deficiency reduced serum IL-6 [32]. Conversely, in Schistosoma-associated PAH, IL-6 was mainly colocalized with the macrophage marker Mac3, suggesting macrophages as another potential source of IL-6 [33]. Classically, NF-κB activation is upstream of IL-6 secretion, and it has been shown to be upregulated in patients with iPAH [34]. However, elevated IL-6 in the lung was not reduced by treatment with the NF-κB inhibitor, pyrrolidine dithiocarbamate, in Sugen-hypoxia-treated rats [35]. Thus, it is likely that multiple cellular sources contribute to elevated IL-6 levels, although the contribution of each cell type may depend on the type of PAH, the severity of the disease, and individual patient differences given the heterogeneous nature of PAH. Furthermore, pulmonary arterial microvascular endothelial cells from patients harboring BMPR2 mutations secreted twice as much IL-6 in response to inflammatory stimuli than control endothelial cells, indicating that the endothelium may also be a significant source of IL-6 in disease [36].

In regard to the cellular mechanisms of IL-6-mediated PH progression, classical IL-6 signaling involves soluble IL-6 binding to its membrane-bound receptor (IL-6R), causing the assembly of a complex involving two molecules each of IL-6, IL-6R and the IL-6 receptor subunit B (gp130) [37]. This complex triggers different signaling pathways, including the JAK–STAT3 pathway, PI3K/AKT pathway, and the MEK/ERK pathway, leading to the expression of pro-inflammatory and pro-survival molecules in the target cell [37]. Interestingly, it has been shown that the treatment of healthy PASMC with IL-6 leads to STAT3 activation, which can cause the further activation of other downstream effectors, including the transcription factor Krüppel-like factor 5 (KLF5) [38]. KLF5 is elevated in both human lung biopsies and cultured human PASMCs isolated from PAH patients and can promote cell proliferation and prevent apoptosis [38]. IL-6 has also been shown to exert its pro-inflammatory effects through the induction of IL-21 expression in Th17 cells and CD4^+^ T cells [39]. In addition, IL-6-mediated STAT3 activation has also been shown to induce the expression of a group of microRNAs (miRNA cluster-17/92) that represses bone morphogenetic protein type (BMPR2) expression, further promoting a pro-proliferative phenotype in vascular cells [40]. Hence, it is likely that IL-6 signaling leads to the downstream activation of multiple pathways centered around cellular pro-inflammatory, pro-proliferative, and anti-apoptotic effects. Importantly, as a stimulatory factor inducing B lymphocyte differentiation into antibody-producing plasma cells, IL-6 production has also been linked to increased immunoglobulin secretion and the production of autoantibodies in PAH [32,41].

#### 2.1.3. IL-18

IL-18, related closely to the IL-1 family of cytokines, is similarly produced in a pro form, and it is processed by caspase-1 [42]. IL-18, largely secreted by macrophages, stimulates a wide variety of pro-inflammatory changes, including the activation of cytotoxic T cells, stimulation of interferon production, and increasing surface expression of adhesion molecules and chemokine production in target cells. IL-18 binding to its receptor complex results in NF-κB activation, and the blockade of IL-18 is presently under intensive investigation as a treatment for inflammatory bowel disease [42]. IL-18 protein is elevated in the plasma of patients with PAH compared with healthy controls [43]. The cellular sources of IL-18 seem to be medial but not intimal SMC of the pulmonary arteries. The IL-18 receptor, IL-18Rα, is expressed in the vascular wall of medial SMC, EC, and infiltrating mononuclear cells [43]. The overexpression of IL-18 in the lungs resulted in mild PAH and RV dilation, but the genetic ablation of IL-18 did not attenuate hypobaric hypoxia-induced PAH and right ventricular hypertrophy [44], suggesting that IL-18 may be a disease modifier, but it is not the causal factor for PAH development. In line with findings documented above with respect to IL-6, IL-18 interacts differently with cells possessing a BMPR2 mutation than those without. Specifically, IL-18 increases the adhesion of monocytes to pulmonary arterial microvascular endothelial cells lacking BMPR2 [45]. However, this interaction did not change endothelial barrier function [45].

##### Chemokines and Their Receptors

The chemokine receptor CCR7 and its ligands have a key role in the homing of T cells and dendritic cells to lymphoid organs [46]. Notably, CCR7 has been found to be downregulated in circulating leukocytes of PAH patients [47]. Substantiating a functional role for this signaling axis, mice lacking CCR7 developed PAH and showed increased perivascular infiltration of leukocytes, consisting mainly of T and B cells [47]. Analogously, the antagonism of CCR7 by CCR7-neutralizing antibodies potentiated PAH, bronchus-associated lymphoid tissue (BALT) formation, and plasma IgG levels in monocrotaline-treated rats [41]. These data suggest that chemokines and their receptors might affect perivascular inflammation by negatively regulating lymphocyte trafficking and BALT formation. As such, CCR7 agonists may bear therapeutic potential in PAH, yet this hypothesis remains to be tested in appropriate model systems.

However, mRNA levels of CCL19 and CCL21, the ligands of CCR7, were not significantly different in lungs of patients with idiopathic PAH as compared to controls [48], although CCL19 is thought to be a sensitive marker for perivascular inflammation in systemic sclerosis. Yet, as CCR ligands are typically expressed in lymphatic vessels and lymphoid organs, they may act locally rather than systemically. Chemokine CXC ligand 13 (CXCL13), which plays an analogous role as CCL19/CCL21-CCR7 for B-cell homing, was elevated in patients with idiopathic PAH and chronic thromboembolic pulmonary hypertension (CTEPH), but there was only a weak association between serum CXCL13 and markers of disease severity and outcome [49]. A possible explanation for these seemingly disparate findings could be that measurements of chemokines in whole lungs or plasma do not reflect their local expression at important sites of disease.

Another important chemokine in the development of PAH is CCR5. CCR5 is expressed in the pulmonary vascular wall and on macrophages, and it has been shown to be upregulated in PAH [50]. In human tissues, CCR5 is found in endothelial cells, smooth muscle, and macrophages in PAH patients, and it is also upregulated following chronic hypoxia in rodent models [50]. Mice deficient in CCR5 were protected from hypoxic PAH and demonstrated a decreased proliferation of PASMC [50]. Elegant experiments using bone marrow chimeras delineated the importance of both parenchymal and leukocyte CCR5 in this process. These data are particularly exciting given the availability of a CCR5 inhibitor currently approved for the treatment of HIV infection. This pathway warrants further clinical study in PAH patients. The crosstalk between macrophage and PASMC CCR5 appears to be synergistic with the CCL2–CCR2 pathway, as a blockade of both pathways in mouse models results in additional benefit when compared to blocking either CCR2 or CCR5 alone [51].

### 2.2. Inflammatory Mediators

#### 2.2.1. Leukotriene B4 (LTB4)

Leukotriene B4 (LTB4) was found to be significantly elevated in the bronchoalveolar lavage fluid of PAH animals and in the blood of PAH patients [52]. Macrophages, expressing high levels of leukotriene A4 hydrolase, the biosynthetic enzyme for LTB4, appear to be the main source of LTB4 [52]. Macrophage-produced LTB4 directly induced the apoptosis of PAEC and the proliferation of PASMC, via a pathway involving endothelial sphingosine kinase 1 and endothelial nitric oxide synthase [52]. In addition, LTB4 enhanced the proliferation, migration, and differentiation of pulmonary artery adventitial fibroblasts in a dose-dependent manner through its cognate G-protein-coupled receptor [53]. LTB4 activated adventitial fibroblasts by upregulating p38 mitogen-activated protein kinase as well as the Nox4-signaling pathway [53]. Blocking LTB4 formation or antagonizing its receptor reversed MCT-induced PAH and prevented PAH-related death, making this seemingly a promising avenue for translational investigation [54]. However, the reversible protease inhibitor Ubenimex (bestatin), which blocks the conversion of LTA4 to LTB4 by the leukotriene A4 hydrolase, failed to demonstrate effectiveness to improve pulmonary vascular resistance or improve exercise capacity in patients with pulmonary arterial hypertension in the Phase 2 LIBERTY study (NCT02664558) [55].

In spite of this trial failing to reach its primary endpoint, promising preclinical studies still implicate LTB4 in PAH pathogenesis in specific subpopulations. A recent study demonstrated that in BMPR2 haploinsufficient rats, the viral transduction of 5-lipoxygenase (5-LO), the enzyme that produces LTB4, results in the development of severe PAH [56]. Of note, BMPR2 mutations are among the most common human mutations found in hereditary PAH, albeit with low penetrance [57]. The transduction of 5-LO resulted in the development of PAH in these rats with similar frequency to humans with BMPR2 mutations. Additionally, the neointimal cells in these animals developed a spontaneous, endogenous expression of non-viral 5-LO, which is a finding that was also seen in patient tissue [56]. Together, these data demonstrate a fundamental interplay between leukotrienes, transforming growth factor (TGF)-β / bone morphogenic protein (BMP) signaling, and the development of PAH. Importantly, these data also suggest that perhaps LTB4-based therapies may show the most promising clinical effectiveness in patients with BMPR2 mutations.

#### 2.2.2. Macrophage Migration Inhibitory Factor (MIF)

Macrophage migration inhibitory factor (MIF), originally identified as a T-cell-derived cytokine that inhibited the random migration of macrophages, has equally been found increased in PAH [58]. MIF is now considered an important pro-inflammatory mediator secreted by numerous cells including T cells, macrophages/monocytes, ECs, and SMCs that can induce in turn the production of cytokines, such as IL-1β, IL-6, and IL-8. In addition, MIF regulates vascular cells through its binding to CD74, which is highly expressed in the endothelium of muscularized pulmonary arterioles and in cultured pulmonary ECs from IPAH patients. Curative treatments with the MIF antagonist ISO-1 or anti-CD74 neutralizing antibodies partially reversed the development of pulmonary hypertension and substantially reduced inflammatory cell infiltration in the rat monocrotaline model of PAH [59]. In addition to its effects on ECs, MIF may act on the proliferation of PASMCs through the activation of the ERK1/2 and JNK pathways in hypoxic pulmonary hypertension [60].

#### 2.2.3. Hypoxia-Induced Mitogenic Factor (HIMF)

Hypoxia-induced mitogenic factor (HIMF) is a well-known marker for alternatively activated (M2) macrophages [61]. HIMF expression in the remodeled pulmonary vasculature positively correlated with increased mean pulmonary arterial pressure [62]. A single systemic injection of recombinant HIMF protein caused early lung inflammation (day 7) and PAH development (day 30) [62]. HIMF stimulates EC activation and apoptosis in the lung via the HIF-1/ vascular endothelial growth factor (VEGF)-A/VEGFR2 signaling pathway [63]. Furthermore, these HIMF-stimulated ECs produce growth factors and chemokines that enhance perivascular immune cell recruitment and SMC growth. In addition, HIMF has been shown to induce expression of the pro-inflammatory cytokine IL-6 in primary lung fibroblasts via the IKK-β/NF-κB/HIF-1 pathway [63].

#### 2.2.4. High Mobility Group Box-1 (HMGB1)

Generally considered to be an atypical cytokine, high mobility group box-1 (HMGB1) is a nuclear molecule that contributes to DNA stability by regulating transcription, repair, and recombination [64]. In response to cellular stress or damage (e.g., hypoxia, infection, sterile inflammation), HMGB1 can be released to the extracellular environment, where it functions as a danger-associated molecular pattern (DAMP) [65], binding multiple receptors, including toll-like receptor (TLR) TLR4, TLR2, and receptor for advanced glycation endproducts (RAGE) [65,66]. HMGB1 was recently shown to be a marker of lytic cell death that was released during cellular rupture following inflammasome activation [66]. HMGB1 levels are elevated in the serum and lungs of PAH patients and animal models of PAH [11,67,68]. Circulating levels of HMGB1 have also been found to moderately correlate with mean pulmonary artery pressure (mPAP) [67]. Histological examination of patients with severe PAH revealed strong extra-nuclear HMGB1 staining in the perivascular adventitia and intima, indicating potentially relevant sites of HMGB1 release [68]. Additionally, endothelial cells isolated from the small pulmonary arteries of patients with idiopathic PAH showed an elevated basal production of HMGB1 and RAGE [69]. Together, these data point to a potentially important role for HMGB1 in the development of PAH.

The pharmacological inhibition of HMGB1 has been an effective strategy for mitigating PAH in several animal models. Treatment of hypoxic mice and MCT-treated rats with an HMGB1 neutralizing antibody can significantly attenuate increases in RVSP and protect against pulmonary vascular remodeling [67,70]. Chronic inhibition of HMGB1 by glycyrrhizin, a natural anti-inflammatory factor that binds HMGB1 directly, also protects against the development of PAH in MCT-treated rats [11]. Similarly, inhibiting HMGB1 receptors has the potential to mitigate PAH. Mice lacking TLR4, one of the main receptors required for pro-inflammatory HMGB1 signaling, are protected against chronic hypoxia-induced PAH [67]. In comparison, the knockdown of RAGE in these mice did not appear to offer the same benefits, suggesting an important role for HMGB1–TLR4 signaling specifically [67]. However, other studies have also found a contribution downstream of RAGE, suggesting that both RAGE and TLR4 may be important for mediating the pro-inflammatory effects of HMGB1 [69,71]. The octapeptide, P5779, which specifically inhibits the interaction between HMGB1 and TLR4, is capable of reversing established disease in Sugen-hypoxia rats as well as improving RVSP, right ventricular dysfunction, and pulmonary vascular remodeling [68]. P5779 was shown to prevent PASMC migration and proliferation, suggesting a potential direct effect of HMGB1 on the lung vasculature [68]. The latter notion is supported by in vitro studies demonstrating a proliferative effect of physiological HMGB1 concentrations on PASMC and EC, presumably via the activation of p38, ERK, and JNK [72]. This potential of P5779 as a promising therapeutic for PAH will be the focus of a future translational investigation.

#### 2.2.5. Complement

The complement cascade forms a crucial element of innate immunity, and it has been implicated in a variety of pulmonary and vascular disorders, including acute lung injury [73]. Recently, an important role for immune complexes and complement activation in PAH has been elucidated [74]. In both human tissue and animal models, the activation of a classical and alternative pathway complement was seen in perivascular lesions [74]. Mice deficient in several elements of the complement cascade were protected from hypoxia-induced perivascular inflammation. Of note, the expression of pro-inflammatory granulocyte monocyte colony stimulating factor (GM-CSF) was found to be downstream of complement activation, as were the proliferative responses of the pulmonary vascular tissues [74]. Additionally, the complement factors C3 and C4 have been identified as PAH biomarkers [75,76], and C3 deficiency partially protects mice from chronic hypoxic PAH, with an associated dampening of immune responses [77]. These results bear particularly important translational value, since complement inhibitors are currently approved and under investigation for the treatment of several conditions, including a variety of glomerular kidney diseases [78].

### 2.3. Immune Cells in PH and Their Roles in Vascular Remodeling

#### 2.3.1. Macrophages

An early and persistent accumulation of macrophages has been observed in perivascular lesions in many patient cohorts and animal models of PAH [2,79,80]. Indeed, a recent study of unbiased computational flow cytometric analysis of human lungs from patients with iPAH and healthy donors demonstrated the profound recruitment of macrophages to isolated pulmonary artery samples [81]. Interventions targeting macrophages have confirmed their role in PAH and pulmonary vascular remodeling. An intratracheal depletion of alveolar macrophages with liposome-encapsulated clodronate attenuated the increase in pulmonary arterial pressure in response to chronic hypoxia in rats [82]. Furthermore, the depletion of macrophages normalized the increase in RVSP seen in BMPR2 knockout mice exposed to chronic hypoxia [83]. To the contrary, an intraperitoneal depletion of macrophages with clodronate liposomes resulted in worsened secondary pulmonary hypertension in a pulmonary fibrosis model; yet, no details about the change of resident alveolar macrophages or circulating macrophages were provided in this study [84]. These data indicate that the subtype and location of macrophages may be a critical factor at play, which is a view that is consistent with the fact that pulmonary macrophage phenotypes change over time in PAH models [85].

Over the past decade, macrophages have emerged as highly heterogeneous cells that can rapidly change their function in response to the local microenvironment. Accordingly, macrophages have been classified into classically (M1) and alternatively (M2) activated phenotypes. While these classifications are fluid and complex, M1 macrophages can be thought to arise during pro-inflammatory states, which are triggered by Toll-like receptor activation and interferons. On the other hand, M2 macrophages are found in more chronic states such as allergy and can be involved in non-resolving inflammation and tissue repair [86]. The pharmacological inhibition or genetic deletion of CX3CR1, which is elevated in the lungs of mice with chronic hypoxic PAH, protected mice against hypoxic PAH [87]. In parallel, the loss of CX3CR1 favored M1 macrophage polarization, and this shift from M2 to M1 abrogated the ability of macrophage-conditioned medium to induce PASMC proliferation in vitro, suggesting a pathophysiological role of CX3CR1 via M2 macrophage polarization in PAH [87]. The kinin B1 receptor, which is expressed on macrophages, was upregulated in the lung tissue of MCT-challenged pneumonectomized rats. Treatment with a specific kinin B1 receptor reduced macrophage counts in bronchoalveolar lavage fluid, as well as CD68^+^ macrophage counts in the perivascular area, suggesting an important role for kinins in monocyte/macrophage recruitment and differentiation [88].

In addition, circulating monocytes can take on an endothelial-like phenotype once adhering to endothelial cells [89,90]. Both macrophages and hyperproliferative endothelial-like cells were observed in plexiform lesions in PAH, and they may indicate the conversion of monocytes to endothelial-like cells that contribute to pulmonary vascular remodeling in PAH. This notion is partially supported by the finding that carboxyfluorescein diacetate-labeled RAW 264.7 macrophages were found retained in the lung vasculature of hypoxic athymic nude mice up to twelve days after injection [91].

#### 2.3.2. Dendritic Cells

Circulating activated myeloid-derived suppressor cells (MDSCs) are significantly increased in PAH patients and correlate with increasing mean pulmonary artery pressure [92]. MDSCs compose a phenotypically diverse subpopulation of cells, of which dendritic cells (DCs) are important components [93]. Belonging broadly to a class of innate lymphoid cells [94], there are at least four main subsets of DCs identified in both mouse and human, including conventional cDC1 and cDC2, plasmacytoid DCs, and monocyte-derived dendritic cells (MoDCs) [95]. Patients with idiopathic PAH had a significant decrease in the number and changes in function of MoDCs [96]. Although the profile of membrane costimulatory molecules of circulating MoDCs in idiopathic PAH was similar to that of control subjects, PAH MoDCs retained higher levels of the T-cell activating molecules CD86 and CD40 after dexamethasone pretreatment. MoDCs from PAH patients induced a stronger activation and proliferation of CD4^+^ T cells, which is associated with a reduced expression of IL-4 (T helper 2 response) and a higher expression of IL-17 (T helper 17 response) [97]. Further work remains to be done to identify the phenotypes and roles of other circulating DCs subsets in PAH.

Upon encountering antigen, immature DC adopts a mature state. Then, the mature DC can activate T and/or B lymphocytes to induce an immunological response. Surprisingly, both in human and experimental PAH, immature DCs accumulate in remodeled pulmonary vessels, and their counts increased with the degree of pulmonary arterial remodeling [98]. As the pulmonary arteries—unlike the airways—are not thought to be frequently exposed to exogenous pathogens, with the important exception of some ultra-fine particles, the recruitment of DCs is likely in response to tissue damage and the release of damage-associated molecular patterns. Such sterile inflammation may potentially link to the development of auto-immunity, as discussed below.

#### 2.3.3. Mast Cells

The accumulation and activation of perivascular mast cells in the lung are prominent histopathological features in idiopathic PAH patients and PAH rats [99,100,101]. Mast cell activation induces the release of various potent molecules via degranulation. The main granule molecules are histamine, serotonin, proteases, lipid mediators, cytokines, and chemokines. Notably, the inhibition of mast cell activation, proliferation, or degranulation has been proven effective to attenuate PAH and pulmonary vascular remodeling in several animal models [99,102,103,104].

Mediators released by mast cell degranulation can interact directly with vascular cells and promote pulmonary vascular remodeling [105]. Mast cell chymase regulates vasomotor tone indirectly, as it can stimulate the regional production of angiotensin II, the activation of endothelin-1, and the secretion of matrix metalloproteases [106,107]. Mast cell-derived tryptase can induce PASMC proliferation and migration, fibroblast proliferation, as well as an enhanced synthesis of fibronectin and matrix metalloproteinase-1 via PAR-2 [105,108]. Mast cells also secrete several isoforms of VEGF, which may regulate the neoangiogenesis observed in PAH [109]. In idiopathic PAH patients, chymase-positive mast cells were located in close proximity to regions with a prominent expression of big-endothelin-1 in the pulmonary vessels [106]. Intervention with the mast cell inhibitors cromolyn and fexofenadine decreased total tryptase levels, and it was accompanied by a drop in VEGF and circulating proangiogenic CD34^+^CD133^+^ progenitor cells, as well as an increase in exhaled nitric oxide [103]. Together, these data suggest that therapies targeting mast cells may be an important translational strategy for PAH treatment, and that this could perhaps be achieved using drugs that are currently available.

Additionally, mast cells sit at a major crossroads for both innate and adaptive immune responses and therefore could control PAH progression and pulmonary vascular remodeling by regulating multiple arms of the immune response. Mast cells-derived IL-6 has been shown to stimulate B cell-related immune responses in the MCT-induced PAH model in rats [32]. Furthermore, mice reconstituted with a human immune system were shown to develop severe PAH in response to chronic hypoxia, and this response could be blunted by anti-mast cell treatment [110]. This result is significant for showing that, first, a factor (or factors) exists specifically in the human immune system that render mice, which normally do not develop severe PAH following chronic hypoxia, susceptible to advanced disease. Second, these results demonstrate a difference in mast cell distribution or function between mice and humans that make mast cell targeting a potential strategy in human disease.

Recently, it has been shown that mast cells play a more complex role in the overall immune response than previously recognized. They can make direct contact with dendritic cells to regulate their antigen-presenting ability and subsequent T cell activation [111]. Increasing evidence demonstrates the interaction between mast cells and T cells in inflammatory models; for instance, mast cells promote the activation, proliferation, and cytokine secretion of CD4^+^ T cells, induce CD8^+^ T cell recruitment, and inhibit regulatory T cell (Treg) activity via secreting histamine and IL-6 [107]. The role of the interaction between mast cells and other immune cells in PAH will require further investigation.

Importantly, the contribution of mast cells to the progression of PAH appears to occur early on in disease development. In MCT-treated rats, the pharmacological inhibition of mast cell degranulation and c-kit from the onset of disease (treatment from day 1 to 21 following MCT injection) significantly attenuated RVSP increase and vascular remodeling; whereas, delayed treatment (from day 21 to 35 post-MCT injection) neither improved hemodynamics nor vascular remodeling [100]. Other effective treatments targeting mast cells in various PAH models were also given preventively in support of this observation [99,104].

#### 2.3.4. T Cells

Two types of tertiary lymphoid tissues (tLT) have been reported in the lungs of PAH patients: perivascular tLT and BALT, which is comprised of B- and T-cell zones with high endothelial venules and dendritic cells. Lymphocyte survival factors and lymphorganogenic cytokines and chemokines were highly expressed in tLT from idiopathic PAH patients [48]. These tLT raise the fascinating possibility of a local adaptive immune response in PAH lungs.

The perivascular infiltration of inflammatory cells in MCT-treated rats is characterized by CD4^+^ T cells [112]. Rag1^−/−^ mice, which are devoid of T and B cells, were protected from the development of pulmonary vascular lesions when exposed to MCT, and the adoptive transfer of T cells from control mice into Rag^−/−^ mice restored vascular injury [112]. Athymic nude rats lacking specifically T cells, given SU5416 alone, without subsequent hypoxia, developed severe PAH and vascular remodeling, which was not observed in euthymic animals [113]. The reconstitution of these animals with immunocompetent splenocytes protected SU5416-treated animals from developing severe PAH, again demonstrating the importance of an intact immune system for the normal progression of PAH [113]. Further reconstitution studies have shown that the specific re-population of T cell-deficient rats with Tregs reduces the exaggerated disease process seen in these animals [114]. This effect may be even more pronounced in females, indicating an important role for Tregs in the sex-specific differences observed in PAH patients [115]. These findings indicate that CD4^+^ Tregs inhibit PAH progression via negatively regulating T cell immune responses and open up an intriguing avenue for cell-based therapies in PAH.

T cell changes in PAH can also be observed in the peripheral blood, although how they relate to immune cell profiles and vascular remodeling processes in the lung remains unclear. Subclass analysis of peripheral blood from patients with idiopathic PAH showed that CD4^+^CD25^high^ Treg cells increased, while CD8^+^ cytotoxic T cells decreased relative to controls [116]. However, others have found no difference in circulating CD4^+^CD25^+^CD127^low^ Treg numbers or in the overall percentage of CD4^+^ T cells between PAH patients and controls [117]. Notwithstanding, circulating Tregs in PAH patients appeared to be dysfunctional, with low levels of pSTAT3, which is a major signaling pathway in Tregs [117]. These findings, namely the differences between pulmonary cell distributions and those in the blood, are in line with data from cancer research showing that abundances of Tregs, CD4^+^, and CD8^+^ T cells differ markedly between tumors and the periphery [118].

#### 2.3.5. B Cells and Autoantibodies in PAH

Similar to T cells, B cells are the main components of tLT in PAH lung tissue, and the overall area taken up by tLT correlates with PAH severity and pulmonary vascular remodeling [41,48]. Rats with B cell deficiency are less susceptible to severe PAH and pulmonary vascular remodeling induced by MCT or Sugen-hypoxia [32]. A recent clinical trial of rituximab (the anti-CD20, B-cell targeted therapeutic antibody) for the treatment of PAH associated with systemic sclerosis (SSc-PAH) demonstrated safety and trends toward efficacy [119]. B cell depletion in SSc-PAH has also been shown to alter the profiles of circulating antibodies [120]. Additionally, several other reports have shown positive results in treating patients with systemic lupus erythematosus (SLE)-PAH with B-cell depletion therapy [121].

Given that B cells are fundamental to the humoral immune response, their role in PAH was mainly thought to be in the regulation of auto-antibody production. Increased autoantibody levels are commonly detected in PAH-associated autoimmune diseases [122,123]. Indeed, lymphoid tissues adjacent to vascular lesions contain germinal centers, indicating that they can locally produce antibodies in a self-sustaining fashion [48]. Serum autoantibodies (anti-RNP, anti-Sm, and antiphospholipid antibody) in SLE/SSc patients were found to be risk factors for the development of PAH [124,125,126,127]. In various PAH animal models, an increased titer of autoantibodies to pulmonary vascular cells was seen following the disease development [32,41]. Injection of control wild-type animals with autoantibodies-containing plasma or enriched IgG was sufficient to produce vascular remodeling and an increase in RVSP, indicating that such auto-antibodies are themselves sufficient to trigger PAH development [41]. Consistently, serum IgG from patients with SSc-PAH and idiopathic PAH has been shown to cause the constriction of PASMC in a collagen matrix [128]. Similarly, the transfer of SSc–IgG-containing autoantibodies into healthy C57BL/6J mice led to more abundant vascular α-smooth muscle actin expression and inflammatory pulmonary vasculopathy [129]. Anti-endothelin receptor type A and anti-Ang receptor type-1 auto-antibodies increased endothelial cytosolic Ca^2+^ concentrations in isolated perfused rat lungs, demonstrating that circulating auto-antibodies are not mere bystanders in PAH but are actually themselves vasoactive and pro-remodeling [129].

#### 2.3.6. Neutrophils

Little attention has so far been paid to neutrophils in the pathogenesis of PAH. In the few published reports, it could be shown that specific proteins expressed by neutrophils, such as myeloperoxidase (MPO) and neutrophil elastase (NE), are elevated in peripheral plasma from PH patients and correlated with the severity of PAH and clinical outcome [130]. Selective NE inhibitors attenuated or even reversed PAH and pulmonary vascular remodeling in the rat model of monocrotaline-induced PAH, and they are the focus of current clinical investigation [131,132]. Even more exciting from a clinical perspective, late intervention with an MPO inhibitor stopped the progression of experimental chronic obstructive pulmonary disease and partially protected against PAH [132].

Recently, the neutrophil/lymphocyte ratio (NLR) has been proposed as a new inflammatory biomarker and can be used as an indicator of systemic inflammation in many diseases, including PAH. In patients with CTEPH or PAH with sarcoidosis, NLR correlated significantly with pulmonary vascular resistance [133,134]. Similarly, NLR correlated with important prognostic biomarkers in PAH patients [135], and high NLR was associated with high morbidity and mortality in patients undergoing thromboendarterectomy [134]. These findings suggest that neutrophils may be more involved in the pathogenesis of PAH than is currently appreciated.

Notably, recent data have linked pulmonary vascular remodeling with the formation of neutrophil extracellular traps (NETs), which play an important role in pulmonary inflammation and autoimmune diseases [136]. NETs are composed of decondensed chromatin fibers coated with granular and cytoplasmic proteins from neutrophils, such as MPO, NE, and α-defensins, and they are released into the extracellular space in response to biochemical, pharmacological, or mechanical stimulation. In addition to being expressed on NET fibers, NE and MPO also regulate NET formation [137]. Increased NETosis has been documented in patients with idiopathic PAH as well as CTEPH with NET-forming neutrophils and extensive areas of NETosis in occlusive plexiform lesions and intrapulmonary thrombi [138]. NETs may contribute to vascular remodeling via different pathways in that they have been shown to induce NF-κB-dependent endothelial angiogenesis in vitro and increased vascularization of matrigel plugs in vivo but also stimulate PASMC proliferation in vitro and the release of endothelin-1 in human PAEC [138].

In addition, neutrophils have the ability to regulate BALT formation, which has been linked to PAH progression [41]. Specifically, the increased propensity of BALB/c mice to form BALT as compared to C57BL/6 mice is associated with neutrophil recruitment [139]. In fact, BALB/c mice have a higher number and percentage of neutrophils than C57BL/6, and the depletion of neutrophils with anti-Ly6G antibody attenuated lipopolysaccharide (LPS)-induced BALT formation [139]. However, so far, there have been no reports of a relationship between neutrophils and BALT in PAH models.

**Figure 2:**
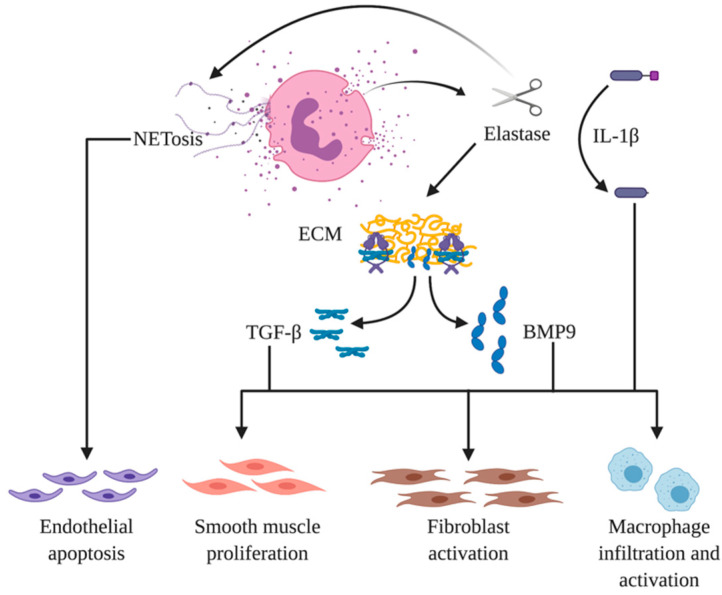
Neutrophil elastase in pulmonary arterial hypertension (PAH). Released from neutrophils, as well as smooth muscle cells, neutrophil elastase influences multiple steps in the pathogenesis of PAH, and it is the subject of significant therapeutic interest. Elastase can degrade the extracellular matrix (ECM), liberating active bone morphogenic protein (BMP9) and transforming growth factor (TGF-β). These cytokines induce phenotypic alterations in smooth muscle cells (SMC), fibroblasts, and macrophages, detailed in the text. Additionally, elastin can cleave and activate interleukin-1β (IL-1β), which is a potent inflammatory cytokine that further stimulates macrophage migration and activation to the pulmonary vasculature. Finally, elastin secretion is involved in the formation of neutrophil extracellular traps (NETs), which themselves can induce endothelial apoptosis, which is a key feature of PAH.

Neutrophils, along with pulmonary vascular smooth muscle cells, are an important source of elastase. Neutrophil elastase is critically involved in lung vascular remodeling, and the inhibition of elastase with small molecule agents or overexpression of its natural antagonist, elafin, reverse PAH in a variety of animal models (Figure 2) [132,140]. In addition to blocking elastase, elafin has been shown to enhance bone morphogenic protein signaling and inhibit NF-κB activity, providing a profound anti-inflammatory effect. A safety and tolerability trial examining elafin as a PAH treatment is currently underway (NCT03522935).

### 2.4. Phenotype Changes of Pulmonary Vascular Cells

Cells residing within the pulmonary vascular wall can serve as both the source of inflammatory mediators as well as their targets. This unique role positions pulmonary vascular cells at a critically important regulatory junction for disease progression and maintenance [141].

#### 2.4.1. Endothelial Cells

The pulmonary artery endothelial cell is thought by most researchers to represent the initial site of disease in PAH, although this is a topic of significant debate [4,142]. Endothelial cells, under various conditions, can themselves serve as non-professional immune cells, synthesizing and secreting inflammatory mediators, as well as being the local targets of inflammation. However, the potential mechanisms of initial endothelial cell activation that may ultimately result in the development of PAH remain unclear. Hypoxia—the cardinal trigger of type III PAH—can decrease endothelial peroxisome proliferator-activated receptor-γ coactivator-1α (PGC-1α), leading to endothelial dysfunction via increased ROS formation, mitochondrial dysfunction, NF-κB activation, and the subsequent secretion of IL-6 and TNF-α [143]. Therefore, upregulating PGC-1α could potentially improve endothelial function and attenuate the inflammatory response in the endothelium [143]. Leptin, which is elevated in idiopathic PAH and SSc-PAH patients, and synthesized by dysfunctional pulmonary endothelium, inhibits Treg proliferation, while enhancing conventional T cell proliferation via modulating T cell autophagy [117,144]. These are only a few examples of endothelial roles in PAH, which have been reviewed extensively elsewhere [145,146].

The most common heritable mutations resulting in PAH, as well as a common locus of acquired mutations, is in bone morphogenetic protein type 2 (BMPR2) [147]. Such mutations may affect the sensitivity of PAEC to inflammatory inputs. Recent studies have shown that BMPR2 deficiency promotes an exaggerated inflammatory response in PAH progression [148]. Challenged with injections of MCT combined with intratracheal instillation of replication-deficient adenovirus expressing 5-lipoxygenase, BMPR2^+/−^ mice developed a sustained increase in RVSP, which was coupled with marked perivascular inflammation of the remodeled vessels and a significantly higher expression of chemokine macrophage inflammatory protein (MIP)-1α and fractalkine receptor in the lung [148]. Along similar lines, acute exposure to LPS increased lung and circulating IL-6 levels in BMPR2^+/−^ mice to a greater degree than in wild-type controls, and chronic LPS administration caused PAH in BMPR2^+/−^ mice but not in wild-type controls [147]. The recruitment of monocytes by PAEC isolated from human carriers of BMPR2 mutations was higher compared to PAEC from non-carriers and from controls, which was likely related to elevated intercellular adhesion molecule-1 (ICAM-1) expression [36]. Such data show the complex interplay between the inflamed, dysfunctional endothelium, which overexpresses adhesion molecules, and the further recruitment of inflammatory cells to diseased vessels.

In addition, PAEC develop a marked pro-inflammatory phenotype in PAH that is evident as as increased expression of ICAM-1, vascular cellular adhesion molecule (VCAM-1), and E-selectin on the endothelium of distal pulmonary arteries in patients with idiopathic PAH [59]. Analogously, freshly isolated human PAEC from idiopathic PAH patients displayed a marked pro-inflammatory transcriptional signature, including elevated expression of IL-1α, IL-6,IL-8, IL-12, MCP-1, E-selectin, ICAM-1, P-selectin and VCAM-1 [59]. In plasma from iPAH patients, P-selectin was found to be increased and thrombomodulin was decreased [149]. Similarly, iPAH was associated with increased plasma and lung levels of CCL2, with PAEC from iPAH patients releasing twice as much CCL2 as did PASMC [150]. These epigenetic changes resulted in a marked increase in monocyte migration, and notably, CCL2-blocking antibodies reduced endothelial chemotactic activity by 60% [150].

#### 2.4.2. Smooth Muscle Cells

PASMC are a cell type that is not terminally differentiated and can retain remarkable plasticity [151]. In Sugen-hypoxia rat models, different αSMA^+^ cells have been detected in vascular lesions: immature SMC (αSMA^+^, SM1^+^, SM2^+/−^, vimentin^+^) in cellular intimal lesions, mature SMC (αSMA^+^, SM1^+^, SM2^+^, vimentin^+/−^) in fibrous intimal lesions, and myofibroblasts (αSMA^+^, SM1^−^, SM2^−^, vimentin^+^) in plexiform lesions [152]. In addition to PASMC, pericytes also contribute to the population of αSMA^+^ cells, and they are abundant in PAH lesions [153,154]. Immature SMC-rich intimal and plexiform lesions were proliferative and were infiltrated by macrophages, while fibrous intimal lesions were less proliferative and were infiltrated by fewer macrophages [152].

With the progression of PAH, changes in signaling pathways in PASMC may alter their pro-inflammatory capabilities. Notably, smooth muscle cells have been shown to relay inflammatory signals in the lung via their ability to secret pro-inflammatory cytokines [155]. P-selectin has been found to be persistently upregulated in the PASMCs of human and hypoxia-induced experimental PAH [156]. Furthermore, there is a marked increase in the expression of phosphorylated, inactive PTEN in the pulmonary vasculature of PAH patients as compared to normal lung tissue [157]. The inactivation of PTEN in PASMC has been previously shown to induce PAH and hypersensitivity to hypoxia [157]. PTEN depletion combined with hypoxia resulted in a synergistic increase in macrophage accumulation and sustained IL-6 production, which may imply that interactions between activated PASMC and macrophages can promote an inflammatory environment via a mutual feed-forward activation loop [157].

#### 2.4.3. Fibroblasts

**Figure 3:**
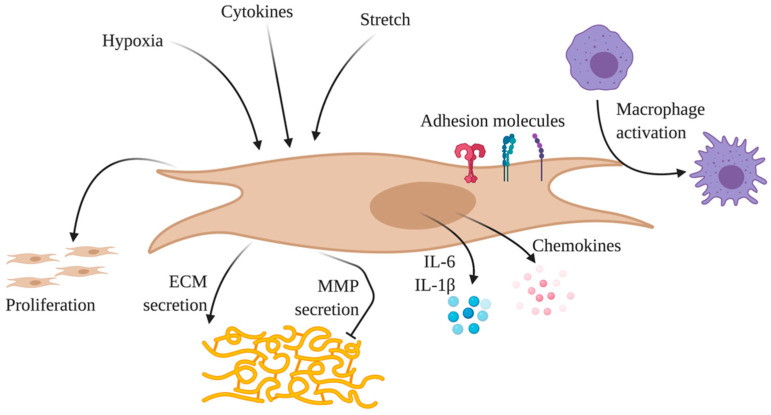
Activated fibroblasts drive PAH pathogenesis. Pulmonary artery adventitial fibroblasts respond to multiple stimuli and then propagate inflammation in PAH. In response to inflammatory cytokines, mechanical stretch, and hypoxia, fibroblasts adopt an activated, pro-inflammatory state. This phenotype is characterized by the overexpression of surface adhesion molecules and other inflammatory surface receptors, and secretion of cytokines and chemokines, importantly IL-6, IL-1β and CCL2. Together, these molecules stimulate both the adhesion and activation of nearby macrophages to propagate the inflammatory response. Activated fibroblasts also alter extracellular matrix homeostasis, secreting ECM proteins, as well as matrix metalloproteinases (MMP) to break down ECM. Finally, fibroblasts themselves proliferate in response to abnormal stimuli encountered in PAH.

Adventitial fibroblasts were originally thought to simply provide mechanical strength to tissues by producing extracellular matrix and providing stromal support. More recently, they were found to be a “sentinel cell” in the vessel wall, responding to various stimuli, such as vascular distension or hypoxia [158]. In response to such stimuli, pulmonary artery adventitial fibroblasts assume a markedly pro-inflammatory phenotype characterized by increased a production of chemokines, cytokines, and adhesion molecules [158]. In addition, interactions between fibroblasts and leukocytes at sites of chronic inflammation appear to promote sustained leukocyte survival and retention resulting in delayed or failed resolution of the inflammatory lesion (Figure 3) [159,160].

Fibroblasts taken from PAH pulmonary artery adventitia display a fundamentally different phenotype compared with those from normal controls [161]. Pulmonary adventitial fibroblasts from a chronic hypoxia model of PAH expressed a persistently pro-inflammatory phenotype, which is defined by a high expression of IL-1β, IL-6, CCL2, CXCL12, CCR7, CXCR4, CD40, CD40L and VCAM-1 [162].

The exposure of naïve bone marrow-derived macrophage (BMDMs) in vitro to intact whole pulmonary artery explants from hypoxia-induced PAH animals significantly increased macrophage activation [161]. However, removal of the adventitia from the PA explant resulted in a marked decrease in transcriptional signatures of activation in BMDM [161]. Exposing naïve BMDMs to conditioned medium generated by adventitial fibroblasts from human idiopathic PAH and hypoxia-induced PAH animals increased the transcription of Cd163, Cd206, Il4ra and Socs3, indicating BMDM activation. These data suggest that activated fibroblasts in the remodeled PA adventitia of animals and humans with PAH provide soluble factors required for macrophage polarization, lending credence to an “outside–in” hypothesis of how inflammation propagates through the vessel wall in PAH [4,161]. Among the possible soluble factors, paracrine IL-6 release may activate macrophages via STAT3, HIF1 and C/EBPβ signaling, independent of IL-4/IL-13-STAT6 and TLR-MyD88 signaling [161].

## 3. Conclusions and Future Directions

In summary, a series of complex interactions between inflammatory cells, vascular cells, and soluble mediators in the lung and periphery promote perivascular inflammation and—presumably—also pulmonary vascular remodeling in PAH (Figure 1). Over the past 15 years, the fundamental role of the immune system in the development of PAH has been increasingly appreciated. The disease is no longer thought to be driven solely by dynamic vasoconstriction, but now inflammation, metabolic processes [163], and even changes akin to oncogenesis [164] have been recognized as critical paradigms in the pathogenesis of PAH. In spite of this new understanding, the majority of our approved clinical therapies for PAH are still vasodilators [3]. Recognition of the importance of inflammation in driving pulmonary vascular remodeling has now spawned a new enthusiasm for clinical and preclinical trials of therapies that go beyond vasodilation to target the cellular and soluble components of the immune system directly, in hopes of slowing or even reversing lung vascular remodeling in this devastating disease. In spite of the wealth of preclinical and clinical data in support of the causal nature of inflammation in the development of PAH, multiple clinical trials investigating immunomodulatory therapy for PAH (discussed above) have been negative or underwhelming. This gives pause to the enthusiasm surrounding this theory of PAH origins and/or progression. Identifying the right patient population and timing of therapy where the largest benefit may be derived represents a critical challenge for the translation of this theory to clinical reality. The extent to which vascular remodeling can be reversed, as well as the reversibility of immune phenomena (such as loss of self-tolerance) seen in PAH, is not clearly known. This leaves open the possibility that patients with severe disease, recruited to clinical trials, will not derive benefit from such therapies. Importantly, these setbacks do not necessarily refute the promise of targeted immunotherapy for the treatment of PAH—it took approximately 4500 years from the first attempts to cause tumor regression via localized infection by Imhotep in 2600 BC to the award of the Nobel Prize in Physiology or Medicine for the discovery of check point inhibitors in 2018. Notably, the revised definition of PAH, which has lowered the minimum mean pulmonary artery pressure for diagnosis from 25 to 20 mmHg, presents a new opportunity to test immunologically-based treatments in patients at an earlier stage of disease [165].
